# Supplementary material for: Metabarcoding dietary analysis of coral dwelling predatory fish demonstrates the minor contribution of coral mutualists to their highly partitioned, generalist diet
Source: PeerJ. 2015 Jun 25;3:e1047. doi: 10.7717/peerj.1047 (PMC4485734; doi:10.7717/peerj.1047)
Supplement: Appendix S1 — BIOCODE reference specimen number or GENBANK accession number are indicated only when sequence similarity with reference barcode sequence was >98% (using BLASTn search) (Machida et al., 2009; Plaisance et al., 2009). When sequence similarity to a reference barcode was <98%, we used the Bayesian assignment tool implemented in SAP to assign each OTU to a higher taxonomic group. Photographs and additional information about BIOCODE reference specimens can be obtained at http://biocode.berkeley.edu. The number of sequences for each OTU is provided in the table. * indicates prey items consisting of >1% of either of the three species diets according to the relative abundance of reads. [file peerj-03-1047-s001.docx]

**Appendix I** – List of Operational Taxonomic Units (OTUs) recovered from fish gut contents. BIOCODE reference specimen number or GENBANK accession number are indicated only when sequence similarity with reference barcode sequence was >98% (using BLASTn search) (Machida et al., 2009; Plaisance et al., 2009). When sequence similarity to a reference barcode was < 98%, we used the Bayesian assignment tool implemented in SAP to assign each OTU to a higher taxonomic group. Photographs and additional information about BIOCODE reference specimens can be obtained at http://biocode.berkeley.edu. The number of sequences for each OTU is provided in the table. * indicates prey items consisting of >1% of either of the three species diets according to the relative abundance of reads.

| **OTU ID** | ***Paracirrhites arcatus*** | ***Neocirrhites armatus*** | ***Caracanthus maculatus*** | **Class** | **Lowest taxon** | **BIOCODE #** | **GENBANK #** |
| --- | --- | --- | --- | --- | --- | --- | --- |
| **X157*** | 241 | 1 | 0 | Polychaeta | Polychaeta | BMOO_02632 |  |
| **X171*** | 0 | 179 | 0 | Polychaeta | Syllidae |  |  |
| **X114** | 127 | 0 | 1 | Polychaeta | Polynoidae | BMOO_17013 |  |
| **X175** | 115 | 0 | 0 | Polychaeta | Autolytinae | BMOO_15029 |  |
| **X193** | 109 | 0 | 0 | Polychaeta | Polychaeta | BMOO_07095 |  |
| **X152** | 0 | 33 | 0 | Polychaeta | Syllidae | BMOO_14091 |  |
| **X3** | 0 | 28 | 0 | Polychaeta | Terebellidae | BMOO-04131 |  |
| **X66** | 0 | 17 | 0 | Polychaeta | Polynoidae | BMOO_14209 |  |
| **X14** | 0 | 5 | 0 | Polychaeta | Polychaeta | XMOO_1226 |  |
| **X307** | 4 | 0 | 0 | Polychaeta | Polychaeta |  |  |
| **X5** | 3 | 0 | 0 | Polychaeta | Nephtyidae |  |  |
| **X212** | 0 | 2 | 0 | Polychaeta | Ampharetidae |  |  |
| **X276** | 0 | 1 | 0 | Polychaeta | Cirratulidae |  |  |
| **X260** | 0 | 1 | 0 | Polychaeta | Notopygos | BMOO_17088 |  |
| **X217** | 1 | 0 | 0 | Polychaeta | Polynoidae |  |  |
| **X219** | 1 | 0 | 0 | Polychaeta | Polychaeta |  |  |
| **X112*** | 0 | 870 | 4 | Malacostraca | Fennera chacei | UF26315 |  |
| **X107*** | 0 | 438 | 109 | Malacostraca | Gammaropsis | BMOO_09698 | HM466409 |
| **X176*** | 0 | 312 | 204 | Malacostraca | Amphilochus | BMOO_10734 |  |
| **X87*** | 18 | 412 | 0 | Malacostraca | Malacostraca |  |  |
| **X163*** | 0 | 366 | 0 | Malacostraca | Santiidae |  |  |
| **X11*** | 0 | 250 | 0 | Malacostraca | Malacostraca |  |  |
| **X140*** | 0 | 240 | 5 | Malacostraca | Isopoda (parasite) | BMOO_01004 |  |
| **X113*** | 239 | 1 | 0 | Malacostraca | Maera | BMOO_10864 | HM466481 |
| **X166*** | 0 | 131 | 0 | Malacostraca | Isopoda | BMOO_01640 |  |
| **X94** | 0 | 0 | 77 | Malacostraca | Munnidae | BMOO_18598 |  |
| **X77** | 0 | 0 | 21 | Malacostraca | Amphipoda |  |  |
| **X73** | 20 | 0 | 0 | Malacostraca | Raoulserenea ornata | BMOO_01078 | HM138806 |
| **X78** | 17 | 0 | 0 | Malacostraca | Bemlos waipio | BMOO_10739 |  |
| **X29** | 14 | 1 | 0 | Malacostraca | Isopoda | XMOO_1242 |  |
| **X68** | 2 | 12 | 0 | Malacostraca | Mysida | BMOO_04351 |  |
| **X169** | 0 | 13 | 0 | Malacostraca | Amphipoda | XMOO_1328 |  |
| **X1** | 0 | 5 | 0 | Malacostraca | Malacostraca |  |  |
| **X16** | 5 | 0 | 0 | Malacostraca | Malacostraca |  |  |
| **X242** | 0 | 4 | 0 | Malacostraca | Isopoda | BMOO_18562 |  |
| **X25** | 1 | 3 | 0 | Malacostraca | Malacostraca |  |  |
| **X316** | 0 | 2 | 0 | Malacostraca | Anthuridea | BMOO-18243 |  |
| **X266** | 1 | 0 | 0 | Malacostraca | Lysiosquillidae | BMOO_04814 |  |
| **X218** | 0 | 1 | 0 | Malacostraca | Tanaidacea | BMOO_18256 |  |
| **X99*** | 0 | 4 | 2047 | Malacostraca | Trapezia areolata | BMOO_01054 | HM465972 |
| **X101*** | 658 | 310 | 36 | Malacostraca | Trapezia serenei | BMOO_01003 |  |
| **X95*** | 726 | 1 | 0 | Malacostraca | Trapezia bidentata | BMOO_01005 | GQ260917 |
| **X148*** | 0 | 4 | 351 | Malacostraca | Trapezia rufopunctata | BMOO_03802 | GQ260918 |
| **X118*** | 190 | 131 | 0 | Malacostraca | Hapalocarcinus | BMOO_01369 |  |
| **X198*** | 0 | 0 | 34 | Malacostraca | Trapeziidae |  |  |
| **X304*** | 0 | 0 | 1 | Malacostraca | Harpiliopsis beaupresii | BMOO_00021 |  |
| **X58*** | 966 | 975 | 0 | Malacostraca | Decapoda |  |  |
| **X181*** | 1651 | 0 | 16 | Malacostraca | Galathea mauritiana | BMOO_01062 | GQ260872 |
| **X75*** | 1462 | 0 | 1 | Malacostraca | Pilodius pugil | BMOO-00480 |  |
| **X119*** | 1293 | 0 | 0 | Malacostraca | Menaethius monoceros | BMOO_00546 |  |
| **X134*** | 1002 | 0 | 0 | Malacostraca | Caridea | plate_M023_F10 | |
| **X83*** | 0 | 0 | 980 | Malacostraca | Phylladiorhynchus integrirostris | BMOO_01548 | GQ260881 |
| **X192*** | 939 | 1 | 15 | Malacostraca | Brachyura larvae | BMOO_11555 | HM465540 |
| **X160*** | 948 | 0 | 0 | Malacostraca | Chlorodiella barbata | BMOO_01186 | HM464355 |
| **X149*** | 894 | 0 | 0 | Malacostraca | Atergatopsis germaini | BMOO_12306 | HM750952 |
| **X109*** | 712 | 0 | 1 | Malacostraca | Liocarpilodes integerrimus | BMOO_01064 | HM465492 |
| **X79*** | 1 | 0 | 661 | Malacostraca | Xanthias lamarcki | BMOO_09901 |  |
| **X47*** | 630 | 0 | 0 | Malacostraca | Petrolisthes | BMOO_01333 | HM466562 |
| **X90*** | 431 | 6 | 167 | Malacostraca | Alpheus dolerus | BMOO_02620 | HM464905 |
| **X115*** | 595 | 0 | 2 | Malacostraca | Pilodius flavus | BMOO_03527 | GQ260927 |
| **X108*** | 458 | 0 | 0 | Malacostraca | Palaemonella tenuipes | BMOO_08993 | JQ180254 |
| **X121*** | 0 | 0 | 422 | Malacostraca | Polynesoecetes kekeae | BMOO_09594 |  |
| **X165*** | 336 | 0 | 1 | Malacostraca | Hippolytidae | BMOO_02247 | GQ260963 |
| **X71*** | 0 | 0 | 288 | Malacostraca | Lachnopodus subacutus | BMOO_03639 |  |
| **X144*** | 249 | 3 | 0 | Malacostraca | Cuapetes ensifrons | BMOO_02313 | HM465055 |
| **X123*** | 251 | 1 | 0 | Malacostraca | Decapoda | BMOO_03281 |  |
| **X72*** | 0 | 0 | 237 | Malacostraca | Calcinus morgani | BMOO_00304 | FJ620281 |
| **X110*** | 0 | 0 | 237 | Malacostraca | Pagurixus | BMOO_11439 | GQ260883 |
| **X139*** | 0 | 184 | 0 | Malacostraca | Decapoda |  |  |
| **X178** | 172 | 0 | 0 | Malacostraca | Palaemonella rotumana | BMOO_02250 | HM466624 |
| **X80** | 125 | 0 | 0 | Malacostraca | Manaethius monoceros | BMOO-01080 |  |
| **X156** | 97 | 0 | 0 | Malacostraca | Palmyria | BMOO_01358 |  |
| **X173** | 84 | 0 | 0 | Malacostraca | Phylladiorhynchus | BMOO_03262 |  |
| **X127** | 83 | 0 | 0 | Malacostraca |  |  |  |
| **X131** | 82 | 0 | 0 | Malacostraca | Perinia tumida | BMOO_01063 | GQ260904 |
| **X186** | 0 | 0 | 69 | Malacostraca | Chlorodiella laevissima | BMOO_01192 | GQ260919 |
| **X136** | 49 | 0 | 0 | Malacostraca | Cyclodius ungulatus | BMOO_03030 | GQ260922 |
| **X82** | 36 | 0 | 0 | Malacostraca | Chlorodiella crispipleopa | BMOO_00481 | JN107923 |
| **X179** | 28 | 0 | 0 | Malacostraca | Palaemonidae |  |  |
| **X155** | 27 | 0 | 0 | Malacostraca | Brachyura larvae | BMOO_11536 |  |
| **X159** | 20 | 0 | 0 | Malacostraca | Actaeinae | BMOO_03228 | GQ260944 |
| **X187** | 14 | 0 | 0 | Malacostraca | Decapoda |  |  |
| **X195** | 13 | 0 | 0 | Malacostraca | Pagurixus | BMOO_11447 | GQ260879 |
| **X199** | 8 | 2 | 0 | Malacostraca | Decapoda |  |  |
| **X135** | 8 | 0 | 0 | Malacostraca | Pilodius | BMOO_02878 | HM465964 |
| **X48** | 7 | 0 | 0 | Malacostraca | Thor | BMOO_11404 | HM466585 |
| **X158** | 6 | 0 | 0 | Malacostraca | Galatheidae | XMOO_0470 | GQ260875 |
| **X33** | 0 | 4 | 0 | Malacostraca | Pagurixus | BMOO_11424 | GQ260885 |
| **X200** | 4 | 0 | 0 | Malacostraca | Decapoda |  |  |
| **X37** | 0 | 3 | 0 | Malacostraca | Alpheidae | DL305B | HM462708 |
| **X49** | 3 | 0 | 0 | Malacostraca | Pandalidae |  |  |
| **X273** | 2 | 0 | 0 | Malacostraca | Huenia | BMOO_03531 |  |
| **X269** | 0 | 0 | 2 | Malacostraca | Paguridae |  |  |
| **X275** | 1 | 0 | 0 | Malacostraca | Jonesius triunguiculatus | BMOO_00869 |  |
| **X243** | 1 | 0 | 0 | Malacostraca | Arete indicus | BMOO_03254 |  |
| **X261** | 0 | 1 | 0 | Malacostraca | Paranamixis fijiensis | BMOO_10322 |  |
| **X292** | 0 | 0 | 1 | Malacostraca | Decapoda |  |  |
| **X299** | 0 | 0 | 1 | Malacostraca | Decapoda |  |  |
| **X230** | 1 | 0 | 0 | Malacostraca | Palaemonidae |  |  |
| **X270** | 1 | 0 | 0 | Malacostraca | Palaemonidae |  |  |
| **X285** | 1 | 0 | 0 | Malacostraca | Decapoda |  |  |
| **X89*** | 0 | 370 | 0 | Maxillopoda | Miraciidae |  |  |
| **X143*** | 3 | 363 | 1 | Maxillopoda | Clausocalanus furcatus | | HM045370 |
| **X182** | 0 | 112 | 0 | Maxillopoda | Maxillopoda |  |  |
| **X145** | 0 | 63 | 1 | Maxillopoda | Siphonostomatoida |  |  |
| **X174** | 0 | 18 | 41 | Maxillopoda | Clausocalanus minor |  | AF462313 |
| **X125** | 0 | 55 | 0 | Maxillopoda | Harpacticoida | BMOO-14006 |  |
| **X31** | 0 | 0 | 50 | Maxillopoda | Lithoglyptidae |  |  |
| **X26** | 35 | 0 | 0 | Maxillopoda | Calanoida |  |  |
| **X18** | 0 | 24 | 0 | Maxillopoda | Maxillopoda |  |  |
| **X45** | 0 | 21 | 0 | Maxillopoda | Calanoida |  |  |
| **X88** | 1 | 20 | 0 | Maxillopoda | Oncaeidae |  |  |
| **X317** | 0 | 13 | 0 | Maxillopoda | Poecilostomatoida |  |  |
| **X39** | 0 | 8 | 0 | Maxillopoda | Harpacticoida |  |  |
| **X15** | 0 | 5 | 0 | Maxillopoda | Harpacticoida |  |  |
| **X309** | 1 | 4 | 0 | Maxillopoda | Oncaeidae |  |  |
| **X305** | 3 | 1 | 0 | Maxillopoda | Acartia negligens |  | EU856813 |
| **X10** | 0 | 3 | 0 | Maxillopoda | Esola | BMOO_17736 |  |
| **X308** | 0 | 3 | 0 | Maxillopoda | Harpacticus | BMOO_18680 |  |
| **X85** | 3 | 0 | 0 | Maxillopoda | Maxillopoda |  |  |
| **X204** | 2 | 0 | 0 | Maxillopoda | Copepoda | BMOO_02761 |  |
| **X311** | 2 | 0 | 0 | Maxillopoda | Corycaeus | BMOO_18376 |  |
| **X231** | 1 | 1 | 0 | Maxillopoda | Oithonidae | BMOO_19003 |  |
| **X233** | 1 | 0 | 0 | Maxillopoda | Temora discaudata |  | EU599555 |
| **X253** | 1 | 0 | 0 | Maxillopoda | Clausocalanus arcuicornis | | GU171292 |
| **X251** | 1 | 0 | 0 | Maxillopoda | Candacia ethiopica |  | HM045297 |
| **X227** | 0 | 1 | 0 | Maxillopoda | Miraciidae |  |  |
| **X237** | 1 | 0 | 0 | Maxillopoda | Maxillopoda |  |  |
| **X254** | 1 | 0 | 0 | Maxillopoda | Verrucidae |  |  |
| **X277** | 1 | 0 | 0 | Maxillopoda | Maxillopoda |  |  |
| **X300** | 1 | 0 | 0 | Maxillopoda | Oncaeidae |  |  |
| **X225** | 0 | 1 | 0 | Gymnolaemata | Margaretta | BMOO_07683 |  |
| **X20** | 0 | 4 | 0 | Sagittoidea | Sagittidae |  |  |
| **X124*** | 1947 | 116 | 0 | Actinopterygii | Stegastes nigricans | MParis0230 | JQ432167 |
| **X105*** | 1745 | 5 | 0 | Actinopterygii | Cirripectes variolosus | MParis0213 | JQ431649 |
| **X141*** | 1144 | 2 | 0 | Actinopterygii | Chromis vanderbilti | MParis0195 | JF434898 |
| **X117*** | 987 | 24 | 0 | Actinopterygii | Gobiidae | FLMOO_1258 |  |
| **X100*** | 467 | 0 | 0 | Actinopterygii | Eviota disrupta | MParis0174 | JQ431734 |
| **X168*** | 317 | 0 | 0 | Actinopterygii | Perciformes |  |  |
| **X52** | 200 | 26 | 0 | Actinopterygii | Nemichthys |  |  |
| **X172** | 3 | 93 | 0 | Actinopterygii | Chromis viridis | MParis523 | FJ583167 |
| **X55** | 83 | 0 | 0 | Actinopterygii | Chaetodon trichrous | MParis608 | JF434814 |
| **X70** | 72 | 0 | 0 | Actinopterygii | Enchelyurus ater | MParis0255 | JQ431707 |
| **X188** | 44 | 0 | 27 | Actinopterygii | Sebastapistes tinkhami | MParis0210 | JQ432148 |
| **X84** | 0 | 38 | 0 | Actinopterygii | Thalassoma hardwicke | Mp0405 | JQ432194 |
| **X150** | 24 | 0 | 0 | Actinopterygii | Gomphosus varius | Mp0407 | JF434980 |
| **X137** | 4 | 0 | 20 | Actinopterygii | Dascyllus flavicaudus | MParis0021 | JQ431678 |
| **X40** | 1 | 15 | 0 | Actinopterygii | Caranx melampygus | Mparis1047 | JF493040 |
| **X56** | 2 | 12 | 0 | Actinopterygii | Scarus psittacus | MBIO890 | JQ432113 |
| **X86** | 10 | 0 | 0 | Actinopterygii | Eviota | Mparis0094 |  |
| **X69** | 0 | 9 | 0 | Actinopterygii | Apogon nigrofasciatus | MParis317 | JQ431447 |
| **X205** | 5 | 4 | 0 | Actinopterygii | Scarus globiceps | MParis600 | JQ432101 |
| **X74** | 1 | 4 | 3 | Actinopterygii | Pseudocheilinus hexataenia | MParis0075 | FJ583970 |
| **X287** | 2 | 4 | 0 | Actinopterygii | Chlorurus sordidus | MParis0314 | JQ431622 |
| **X67** | 6 | 0 | 0 | Actinopterygii | Pomachromis fuscidorsalis | MParis650 | JF435137 |
| **X313** | 5 | 0 | 0 | Actinopterygii | Dascyllus aruanus | Mp0390 | JQ431674 |
| **X60** | 0 | 5 | 0 | Actinopterygii | Amblycirrhitus bimacula | MParis0858 | JQ431404 |
| **X61** | 0 | 4 | 0 | Actinopterygii | Plectroglyphidodon dickii | | JQ350224 |
| **X297** | 0 | 2 | 0 | Actinopterygii | Decapterus macarellus | FLMOO_848 | JF493337 |
| **X264** | 2 | 0 | 0 | Actinopterygii | Plectroglyphidodon johnstonianus | MParis0050 | JQ432008 |
| **X310** | 2 | 0 | 0 | Actinopterygii | Halichoeres hortulanus | MParis0062 | FJ583505 |
| **X286** | 2 | 0 | 0 | Actinopterygii | Ctenochaetus striatus | Mparis0090 | HM034214 |
| **X282** | 1 | 0 | 0 | Actinopterygii | Zebrasoma scopas | MParis0106 | HM034286 |
| **X288** | 0 | 1 | 0 | Actinopterygii | Cheilopogon pitcairnensis | MParis0144 | JQ431616 |
| **X257** | 0 | 1 | 0 | Actinopterygii | Gymnothorax buroensis | MParis290 | JQ431790 |
| **X280** | 0 | 1 | 0 | Actinopterygii | Chromis margaritifer | MParis739 | FJ583158 |
| **X268** | 1 | 0 | 0 | Actinopterygii | Caranx sexfasciatus | MParis764 | HQ560966 |
| **X185** | 0 | 56 | 0 | Ascidiacea | Enterogona |  |  |
| **X220** | 0 | 1 | 0 | Ascidiacea | Didemnidae |  |  |
| **X103*** | 0 | 2814 | 3246 | Anthozoa | Pocillopora | BMOO_02442 | AY139813 |
| **X30** | 0 | 5 | 0 | Anthozoa | Bunodeopsis medusoides | BMOO_09018 |  |
| **X153*** | 0 | 0 | 174 | Hydrozoa | Halopteris constricta | BMOO_08729 |  |
| **X180** | 0 | 19 | 0 | Hydrozoa | Plumularia | BMOO_08101 |  |
| **X34** | 7 | 0 | 0 | Hydrozoa | Agalmatidae |  |  |
| **X201** | 1 | 0 | 0 | Hydrozoa | Hydrozoa |  |  |
| **X247** | 1 | 0 | 0 | Dinophyceae | Azadinium poporum |  | FJ217817 |
| **X312** | 3 | 0 | 0 | Echinoidea | Echinoidea | BMOO_03691 |  |
| **X130*** | 254 | 0 | 0 | Ophiuroidea | Ophiactis | XMOO_0453 | GU480578 |
| **X120** | 1 | 86 | 7 | Ophiuroidea | Ophiocoma pica | DL195 |  |
| **X267** | 1 | 0 | 0 | Prymnesiophyceae | Emiliania huxleyi |  | AY342361 |
| **X23** | 4 | 0 | 0 | Enteropneusta | Ptychoderidae |  |  |
| **X35** | 6 | 0 | 0 | Phaeophyceae | Sargassaceae |  |  |
| **X146** | 0 | 93 | 0 | Cephalopoda | Argonautidae |  |  |
| **X116*** | 110 | 0 | 2358 | Gastropoda | Pascula muricata | BMOO_01709 | FR853840 |
| **X97*** | 3 | 0 | 2078 | Gastropoda | Drupa ricinus | BMOO_02024 | EU391571 |
| **X132*** | 0 | 0 | 787 | Gastropoda | Lienardia mighelsi | BMOO_02576 |  |
| **X129*** | 0 | 777 | 0 | Gastropoda | Dendropoma | BMOO_09729 |  |
| **X197*** | 0 | 249 | 0 | Gastropoda | Viriola incisa | BMOO_03122 |  |
| **X142*** | 0 | 180 | 0 | Gastropoda | Cerithiidae | BMOO_04306 |  |
| **X122*** | 0 | 160 | 0 | Gastropoda | Vitricithna marmorata | BMOO_02586 |  |
| **X162*** | 0 | 140 | 0 | Gastropoda | Cerithiidae |  |  |
| **X59** | 0 | 79 | 0 | Gastropoda | Phenacolepadidae | BMOO_05161 |  |
| **X102** | 0 | 75 | 0 | Gastropoda | Petalifera | BMOO_09252 |  |
| **X64** | 0 | 46 | 0 | Gastropoda | Gastropoda |  |  |
| **X65** | 0 | 18 | 0 | Gastropoda | Homalopoma maculosa | BMOO_03487 | AY923944 |
| **X81** | 0 | 14 | 0 | Gastropoda | Cerithium | BMOO_07780 |  |
| **X256** | 0 | 5 | 0 | Gastropoda | Haminoea natalensis | BMOO_09273 |  |
| **X41** | 4 | 0 | 0 | Gastropoda | Stylocheilus striatus | BMOO_00533 | AF156156 |
| **X262** | 0 | 4 | 0 | Gastropoda | Stomatolina rubra | BMOO-18165 |  |
| **X51** | 4 | 0 | 0 | Gastropoda | Dendropoma |  |  |
| **X226** | 0 | 2 | 0 | Gastropoda | Dendropoma gregaria | BMOO_01542 |  |
| **X298** | 1 | 0 | 0 | Gastropoda | Monetaria caputdraconis | BMOO_00989 | AY161643 |
| **X265** | 0 | 1 | 0 | Gastropoda | Cerithiidae | BMOO_04307 |  |
| **X306** | 1 | 0 | 0 | Gastropoda | Monetaria annulus | BMOO_09567 | DQ324060 |
| **X221** | 1 | 0 | 0 | Gastropoda | Dolabrifera dolabrifera | DL328 | AF156149 |
| **X278** | 1 | 0 | 0 | Gastropoda | Gastropoda |  |  |
| **X289** | 1 | 0 | 0 | Gastropoda | Vanikoridae |  |  |
| **X53** | 0 | 8 | 0 | Secernentea | Aphelenchoididae |  |  |
| **X202** | 2 | 0 | 0 | Secernentea | Camallanidae |  |  |
| **X2*** | 21 | 0 | 222 | Cestoda | Cestoda |  |  |
| **X177** | 26 | 0 | 50 | Cestoda | Cestoda |  |  |
| **X12** | 12 | 0 | 0 | Trematoda | Trematoda |  |  |
| **X229** | 1 | 0 | 0 | Turbellaria | Turbellaria | BMOO_02756 |  |
| **X138*** | 1 | 188 | 0 | Demospongiae | Demospongiae |  |  |
| **X279** | 0 | 2 | 0 | Demospongiae | Coelosphaeridae |  |  |
| **X281** | 0 | 1 | 0 | Demospongiae | Clionaidae |  |  |
| **X252** | 0 | 0 | 1 | Compsopogonophyceae | Erythrotrichiaceae |  |  |
| **X151** | 107 | 0 | 0 | Florideophyceae | Gigartinales |  |  |
| **X54** | 3 | 0 | 0 | Florideophyceae | Ceramiales |  |  |
| **X283** | 0 | 0 | 2 | Florideophyceae | Titanoderma prototypum | | HQ423070 |
| **X293** | 2 | 0 | 0 | Florideophyceae | Gelidiaceae |  |  |
| **X274** | 0 | 1 | 0 | Florideophyceae | Corallinaceae |  |  |
| **X294** | 0 | 1 | 0 | Florideophyceae | Corallinaceae |  |  |
| **X147*** | 0 | 251 | 0 | N/A | Sipuncula | BMOO_02829 |  |
| **X236** | 0 | 2 | 0 | N/A | Sipuncula | BMOO_02415 |  |
| **X96*** | 20 | 296 | 907 | Unidentified | Unidentified |  |  |
| **X93*** | 587 | 0 | 10 | Unidentified | Unidentified |  |  |
| **X38*** | 1 | 548 | 0 | Unidentified | Unidentified |  |  |
| **X133*** | 0 | 446 | 0 | Unidentified | Unidentified |  |  |
| **X196*** | 0 | 167 | 0 | Unidentified | Unidentified |  |  |
| **X104** | 0 | 101 | 0 | Unidentified | Unidentified |  |  |
| **X91** | 0 | 0 | 91 | Unidentified | Unidentified |  |  |
| **X42** | 83 | 0 | 0 | Unidentified | Unidentified |  |  |
| **X24** | 0 | 52 | 0 | Unidentified | Unidentified |  |  |
| **X6** | 0 | 42 | 0 | Unidentified | Unidentified |  |  |
| **X154** | 0 | 30 | 0 | Unidentified | Unidentified |  |  |
| **X183** | 9 | 10 | 9 | Unidentified | Unidentified |  |  |
| **X189** | 0 | 25 | 0 | Unidentified | Unidentified |  |  |
| **X13** | 0 | 21 | 0 | Unidentified | Unidentified |  |  |
| **X27** | 0 | 16 | 0 | Unidentified | Unidentified |  |  |
| **X9** | 0 | 0 | 13 | Unidentified | Unidentified |  |  |
| **X22** | 12 | 0 | 0 | Unidentified | Unidentified |  |  |
| **X194** | 0 | 11 | 0 | Unidentified | Unidentified |  |  |
| **X46** | 0 | 10 | 0 | Unidentified | Unidentified |  |  |
| **X62** | 0 | 10 | 0 | Unidentified | Unidentified |  |  |
| **X43** | 0 | 0 | 10 | Unidentified | Unidentified |  |  |
| **X4** | 0 | 7 | 0 | Unidentified | Unidentified |  |  |
| **X167** | 7 | 0 | 0 | Unidentified | Unidentified |  |  |
| **X19** | 0 | 0 | 6 | Unidentified | Unidentified |  |  |
| **X36** | 0 | 5 | 0 | Unidentified | Unidentified |  |  |
| **X63** | 0 | 5 | 0 | Unidentified | Unidentified |  |  |
| **X314** | 3 | 2 | 0 | Unidentified | Unidentified |  |  |
| **X7** | 0 | 4 | 0 | Unidentified | Unidentified |  |  |
| **X28** | 0 | 3 | 0 | Unidentified | Unidentified |  |  |
| **X32** | 0 | 3 | 0 | Unidentified | Unidentified |  |  |
| **X214** | 0 | 3 | 0 | Unidentified | Unidentified |  |  |
| **X258** | 0 | 3 | 0 | Unidentified | Unidentified |  |  |
| **X245** | 0 | 0 | 3 | Unidentified | Unidentified |  |  |
| **X208** | 0 | 2 | 0 | Unidentified | Unidentified |  |  |
| **X210** | 0 | 2 | 0 | Unidentified | Unidentified |  |  |
| **X222** | 0 | 2 | 0 | Unidentified | Unidentified |  |  |
| **X223** | 0 | 2 | 0 | Unidentified | Unidentified |  |  |
| **X232** | 0 | 2 | 0 | Unidentified | Unidentified |  |  |
| **X244** | 0 | 2 | 0 | Unidentified | Unidentified |  |  |
| **X250** | 0 | 2 | 0 | Unidentified | Unidentified |  |  |
| **X319** | 0 | 2 | 0 | Unidentified | Unidentified |  |  |
| **X296** | 2 | 0 | 0 | Unidentified | Unidentified |  |  |
| **X315** | 2 | 0 | 0 | Unidentified | Unidentified |  |  |
| **X207** | 0 | 1 | 0 | Unidentified | Unidentified |  |  |
| **X211** | 0 | 1 | 0 | Unidentified | Unidentified |  |  |
| **X228** | 0 | 1 | 0 | Unidentified | Unidentified |  |  |
| **X234** | 0 | 1 | 0 | Unidentified | Unidentified |  |  |
| **X235** | 0 | 1 | 0 | Unidentified | Unidentified |  |  |
| **X241** | 0 | 1 | 0 | Unidentified | Unidentified |  |  |
| **X249** | 0 | 1 | 0 | Unidentified | Unidentified |  |  |
| **X259** | 0 | 1 | 0 | Unidentified | Unidentified |  |  |
| **X215** | 0 | 0 | 1 | Unidentified | Unidentified |  |  |
| **X216** | 0 | 0 | 1 | Unidentified | Unidentified |  |  |
| **X224** | 0 | 0 | 1 | Unidentified | Unidentified |  |  |
| **X240** | 0 | 0 | 1 | Unidentified | Unidentified |  |  |
| **X246** | 0 | 0 | 1 | Unidentified | Unidentified |  |  |
| **X248** | 0 | 0 | 1 | Unidentified | Unidentified |  |  |
| **X271** | 0 | 0 | 1 | Unidentified | Unidentified |  |  |
| **X295** | 0 | 0 | 1 | Unidentified | Unidentified |  |  |
| **X301** | 0 | 0 | 1 | Unidentified | Unidentified |  |  |
| **X302** | 0 | 0 | 1 | Unidentified | Unidentified |  |  |
| **X203** | 1 | 0 | 0 | Unidentified | Unidentified |  |  |
| **X238** | 1 | 0 | 0 | Unidentified | Unidentified |  |  |
| **X239** | 1 | 0 | 0 | Unidentified | Unidentified |  |  |
| **X263** | 1 | 0 | 0 | Unidentified | Unidentified |  |  |
| **X290** | 1 | 0 | 0 | Unidentified | Unidentified |  |  |
| **X291** | 1 | 0 | 0 | Unidentified | Unidentified |  |  |
